# Experience of distance education for project-based learning in data science

**DOI:** 10.1007/s42081-022-00154-2

**Published:** 2022-04-09

**Authors:** Kentaro Sakamaki, Masataka Taguri, Hiromu Nishiuchi, Yoshitomo Akimoto, Kazuyuki Koizumi

**Affiliations:** 1 Center for Data Science, Yokohama City University, 22-2 Seto, Kanazawa-ku, Yokohama, 236-0027 Japan; 2 Department of Data Science, Graduate School of Data Science, Yokohama City University, Yokohama, Japan

**Keywords:** Data science, Project-based learning, Problem-based learning, Distance education, Society 5.0

## Abstract

Data science plays an important role in many fields. Project-based learning is an effective teaching approach because students can learn data science practices based on real-world problems and real-world data. Because of a pandemic of COVID-19, we provided project-based learning as distance education (synchronic distance education). In this study, we explain how we developed and conducted it and provide survey results from students. The survey showed about 30% of the students found it difficult to communicate with each other and with teachers. However, it suggested that they could communicate to some extent even by remote group work. We found that, in remote communication, it is important to see the faces of all the students (and teachers) on the Zoom screen when they discuss using screen sharing. There remain some challenges such as timing to start talking and casual questions to teachers. Although some issues should be improved, distance education for project-based learning in data science can be implemented effectively.

**Supplementary Information:**

The online version contains supplementary material available at 10.1007/s42081-022-00154-2.

## Introduction

**Fig. 1:**
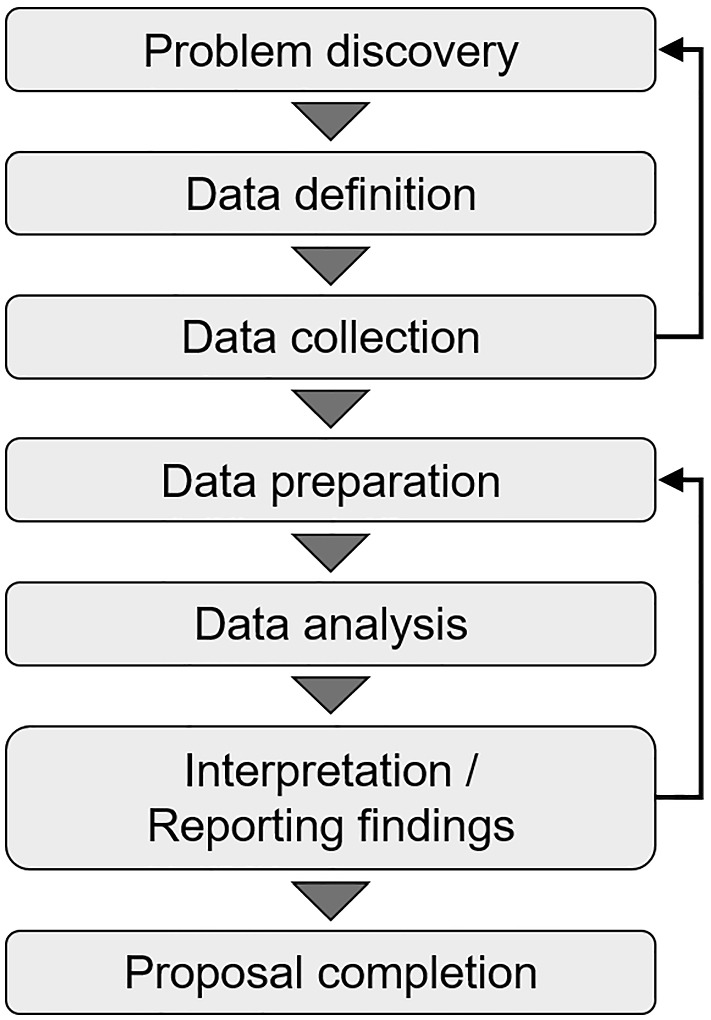
Process for applying data science to real problems

Data science skills are important in a data-driven society that requires data analysis skills, data utilization skills, cross-disciplinary knowledge, and advanced expertise (Cabinet Office & Government of Japan, 2020). Data science consists of elements of statistics, computer science, and a human perspective (Blei & Smyth, 2017). Although data science education should include elements of statistical education, it has been pointed out that conventional statistical education is not sufficient for data science education (Blei & Smyth, 2017; Hardin et al., 2015; Wickham et al., 2018). In data science education, it should be considered that some data science skills can be acquired through collaboration and experience with others with diverse backgrounds (Blei & Smyth, 2017). In practice, it becomes necessary to collaborate with a wider variety of specialists for solving problems as the data diversity and data size increase (Yamaguchi et al., 2020). Therefore, in data science education, it is advisable to acquire skills such as communication skills and leadership in addition to data analysis skills. One of the methods for practical data science education is project-based learning (PBL). In D-STEP (YOKOHAMA Data Scientist Educational Program), Yokohama City University provides PBL where students can experience the process of data science, as shown in Fig. 1. In particular, through group work in collaboration with students with various backgrounds, they learn the skills related to understanding the background of the real problem, setting the appropriate problem for solving with data, and proposing a solution to the problem.

Although PBL with group work is often conducted as face-to-face education, distance education should be conducted instead of face-to-face education due to the spread of COVID-19 infection. In addition, regardless of COVID-19, there is a need for distance education of data science in Japan because about 60% of universities lack teachers who can take charge of data science education, including PBL (Japan Inter-University Consortium for Mathematics Data Science Education, 2021). Therefore, it is important to develop PBL with group work for data science education as distance education. Studies on distance education have been summarized by several meta-analyses and systematic reviews (Carrillo & Flores, 2020; Ebner & Gegenfurtner, 2019; Gegenfurtner & Ebner, 2019; Martin et al., 2020; Means et al., 2009). In the meta-analysis of Ebner et al. (Ebner & Gegenfurtner, 2019) and Gegenfurtner et al. (Gegenfurtner & Ebner, 2019), synchronous distance education rather than face-to-face and asynchronous distance education had a higher learning effect. However, they did not deal with PBL. Other studies, such as Means et al. (Means et al., 2009) and Carrillo and Flores (Carrillo & Flores, 2020), deal only with PBL using asynchronous distance education. As Bonk and Wiley (Bonk & Wiley, 2020) point out, there is currently a growing interest in synchronous distance education using tools such as Zoom, Cisco WebEx, and Google Hangouts. Maher (Maher, 2020) shows the usefulness of Zoom in synchronous distance education for teaching practice. However, to our best knowledge, there is no study of synchronous distance education for PBL with group work, especially for data science education.

When developing synchronous distance education for PBL with group work using tools such as Zoom, problems for communication should be verified. For example, it is difficult to see hand gestures and other body languages, and some fatigue is caused just by looking at the face (Sklar, 2020). In addition, although data analysis can be conducted on an individual laptop, it is difficult to share data analysis results and other information while looking at the face for discussion. There are some problems handled differently than in face-to-face education, such as the confidentiality of the data.

In this study, we research the feasibility and problems of distance education for data science education from the practice of synchronous distance education of PBL and questionnaire survey in 2020. In consideration of the characteristics of PBL in data science education and the experiences of existing studies, we evaluate the feasibility of group work focusing on communication. In addition, to confirm the usefulness of distance education shown in the existing studies, we also survey styles (face-to-face or distance education) for lectures and presentations other than group work that compose PBL.

## Project-based learning in Yokohama City University in 2020

### Course overview

Our PBL is conducted for the first year of the master's program. In this study, one of two PBL courses was introduced. We had a total of 15 classes and met online twice a week for eight weeks. Each class meeting lasts 90 min. In 2020, the project for PBL was to reduce the amount of uncollected delinquent municipal tax. In our course, we provided practical education using group work for client interviews (simulated interviews), analysis of individual data (pseudo data) of delinquents, the proposal of problem solutions, and presentation. One of the purposes of group work was a collaboration with students who have different specialties. The contents of the 15 classes are shown in Table 1.

**Table 1:** Classes for problem-based learning in 2020

1	Lecture 1: Tips for data analysis
2	Lecture 2: Logical thinking
3	Exploration of project
4	Lecture 3: Data analysis / R programming
5	Lecture 4: Tips for interview knowledge / simulated interview
6	Group work 1: Task setting / data analysis
7	Group work 2: Data analysis / presentation material creation
8	Interim presentation
9	Lecture 5: Building a logic for solution proposal of problem solution
10	Group work 3: Reexamination based on the review in the interim presentation
11	Group work 4: Data analysis / presentation material creation
12	Group work 5: Data analysis / presentation material creation
13	Rehearsal for final presentation
14	Group work 6: Data analysis / presentation material creation
15	Final presentation

The project was based on consultation from an existing local government. Many local governments have the problem that while spending on welfare and economic policies increases, income from taxes and social insurance premiums decreases, and delinquency in municipal taxes has become a problem. We also considered key performance indicators and approaches to delinquents based on the consultation. To clarify these issues, the simulated interviews were made from the consultation. From the viewpoint of personal information protection, it is difficult for local governments to provide real data. Therefore, we used pseudo data created based on historical data from the consultation. Although there were simulated parts in our PBL, students were able to experience the process practically, as shown in Fig. 1.

Group work, lectures, and presentations were all conducted online (distance education). In each group work, students discussed interview contents, task setting, the results of literature search, the direction of data analysis, interpretation of analysis results, and proposal of problem solution. In some situations, literature search and data analysis were performed individually. Unlike face-to-face education, it was difficult to efficiently share information on multiple tasks at the same time for group work. Group work outside of the lecture hours was also conducted online. Therefore, we conducted distance education using some tools to deal with these issues.

### Tools for distance education for project-based learning

To conduct PBL as distance education, we used five tools in the following.

(1) Zoom.

**Fig. 2:**
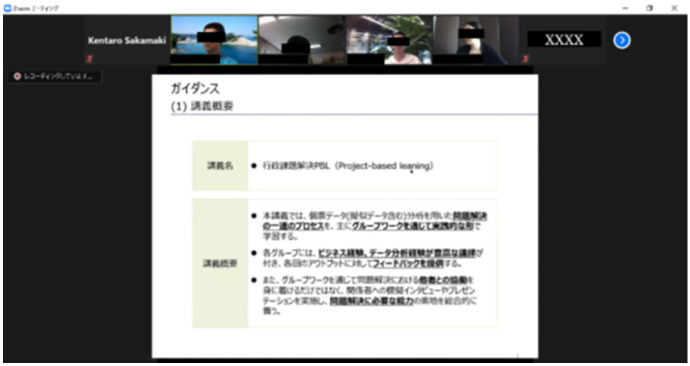
Zoom screen

The Zoom was used for group work, lectures, and presentations. Figure 2 shows an example of the Zoom screen. In group work, the breakout room function was used. Teachers went around the breakout rooms to check the direction of discussions, and students returned to the main session with the teacher and had a direct dialogue if they had questions. These were conducted in consideration of a face-to-face manner.

(2) Teams.

Teams provided by Microsoft was used to save the materials created in group work, to contact students and teachers, and to perform group work outside of the lecture hours. Teams were created for each group. Teams was also used to confirm the progress of group work and presentation materials by teachers.

(3) Learning management system (LMS).

Since D-STEP also conducts lectures other than PBL, D-STEP has an original LMS. LMS was used for sharing information such as Zoom address and lecture materials, confirmation of attendance, and submission of assignments. When new materials were needed during the lecture, they were distributed in LMS. We put the recorded lecture in LMS for the review or convenience of those who had missed it.

(4) Azure notebooks.

Azure, a cloud service provided by Microsoft, was used for R (or Python) execution. Remote support was difficult if problems occurred in R or RStudio installation, package installation, or programming. Therefore, we used Azure Notebooks to support remote programming.

(5) VPN connection.

To search articles for clarifying issues and proposing solutions to problems, we used a VPN connection to download articles from contracted journals.

### Group work for distance education

To facilitate group work in distance education, each group was composed of 4 or 5 students in consideration of role sharing. In addition, we created groups based on the students’ backgrounds so that each student with different background could collaborate. Since there were seven working students out of 37 students, we decided to have one working student in 7 out of 8 groups.

Unlike face-to-face education conducted in one lecture room, it was difficult for teachers to take a view of the whole groups at the same time in distance education. Therefore, a leader was determined in each group and the leader reported progress during and at the end of the class. To encourage each student to voluntarily participate in group work, the final presentation was conducted in a competition format.

## Questionnaire survey

To evaluate the feasibility of PBL by distance education, we conducted a questionnaire survey of 37 students using Forms provided by Microsoft. The deadline for the questionnaire survey was one week after the end of the final class. The questionnaire items are shown in Table 2.

**Table 2:** Questionnaire items

Q1	Communication between students in discussions using Zoom
Q2	Communication with teachers in discussions using Zoom
Q3	Points designed to facilitate group work
Q4	What number of students is appropriate as one group in remote group work
Q5	Reason for Q4
Q6	Useful tool for group work and distance education
Q7	Desirable style of lecture
Q8	Reason for Q7
Q9	Desirable style of group work
Q10	Reason for Q9
Q11	Desirable style of presentation
Q12	Reason for Q11
Q13	Good points in distance education
Q14	Points to be improved (bad points) in distance education

Questions 1, 2, 7, 9, 11 were multiple choices (single answer), Question 6 was multiple choices (multiple answers allowed), and Questions 3, 4, 5, 8, 10, 12, 13, 14 were free text. The choices for Questions 1 and 2 were “easy”, “somewhat easy”, “neither easy nor difficult”, “somewhat difficult”, or “difficult”. The choices for question 6 were “Zoom”, “Teams”, “LMS”, “Azure Notebooks”, “VPN connection”, and “Other (free text)”. The choices for Questions 7, 9, and 11 were “distance education”, “face-to-face education”, and “both are fine”. In addition, answer to Questions 1, 2, 4, 6, 7, 9, and 11 was mandatory, and other questions were free-answer.

For multiple-choice questions, the answers were summarized by frequency and percentage. For open-ended questions, answers that appeared multiple times were selected.

## Survey results

### Questions regarding communication (Q1, 2, and 3)

The summary of the answers to Question 1 (communication between students) is as follows; “easy”: 8 (21.6%), “somewhat easy”: 13 (35.1%), “neither easy nor difficult”: 4 (10.8%), “somewhat difficult”: 8 (21.6%), “difficult”: 3 (8.1%), and unanswered: 1 (2.7%). The summary of the answers to Question 2 (communication with teachers) is as follows; “easy”: 4 (10.8%), “somewhat easy”: 11 (29.7%), “neither easy nor difficult”: 9 (24.3%), “somewhat difficult”: 10 (27.0%), “difficult”: 2 (5.4%), and unanswered: 1 (2.7%). As shown in Supplementary Table 1, answers in Q1 and Q2 were related.

The points that the students designed to facilitate group work (Question 3) were sharing roles, showing your face on Zoom, using communication tools, and respecting each other's opinions. More specifically, to eliminate the difficulty of communicating using Zoom, they considered speaking a little more positively than face-to-face, reacting as loudly as possible, and devising the timing to start speaking. Some groups used Slack other than Teams, but some found it confusing to use a variety of communication tools. In group work outside of the lecture hours, synchronic communication was difficult for some groups because the activity time of group members was different.

### Questions regarding group work (Q4, 5, and 6)

The summary of the answers to Question 4 (the number of students in one group) was as follows; 2 students: 1 (2.7%), 3 students: 2 (5.4%), 4 students: 19 (51.4%), 5 students: 12 (32.4%), 6 students: 2 (5.4%), unanswered: 1 (2.7%). Those who answered "easy" or "somewhat easy" in Q1 or Q2 answered larger numbers (Supplementary Table 2). The reasons for answering 4 or 5 students in Question 5 were as follows; faces of students and teachers could be seen on Zoom while sharing the screen (Fig. 1), it was easier to exchange opinions with a smaller number of students, it was easier to have a sense of ownership, and a group consisted of students with various backgrounds.

The summary of the answers to Question 6 (the useful tools) as follows; “Zoom”: 33 (89.2%), “Teams”: 29 (78.4%), “Portal”: 13 (35.1%), “Azure Notebooks”: 9 (24.3%), “VPN connection”: 1 (2.7%), “Others (Slack)”: 4 (10.8%).

### Questions regarding style of classes (Q7–12)

The summary of the answers to Question 7 (desirable style of lecture) was as follows; “distance education”: 19 (51.4%), “face-to-face education”: 6 (16.2%), “both are fine”: 11 (29.7%), unanswered: 1 (2.7%). Those who answered "easy" or "somewhat easy" in Q1 or Q2 selected "distance education" more (Supplementary Table 3). In reasons for choosing “distance education” (Question 8), in addition to reducing commuting time, taking classes at any place, being able to take classes regardless of physical condition, many students answered that lectures by distance education are no different from face-to-face education. Many of the reasons for choosing “face-to-face education” (Question 8) were the ease of asking questions to teachers individually.

The summary of the answers to Question 9 (desirable style of group work) was as follows; “distance education”: 5 (13.5%), “face-to-face education”: 22 (59.5%), “both are fine”: 9 (24.3%), unanswered: 1 (2.7%). Almost all those who answered "difficult" or "somewhat difficult" in Q1 or Q2 selected "face-to-face education" (Supplementary Table 4). The reasons for choosing “distance education” or “both are fine” (Question 10) were as follows; there was active discussion even remotely, data analysis could be performed remotely, screen sharing was successful, and some had experiences of online meetings at the company. Although some students chose “distance education” in Question 9, they answered that face-to-face education is better for brainstorming in Question 10. Most of the reasons for choosing “face-to-face education” (Question 10) were communication: the importance of nonverbal communication in reading intent, casual communication using whiteboards and memos, and that it is easy to check the progress of individual work when the roles are shared.

The summary of the answers to Question 11 (desirable style of presentation) was as follows; “distance education”: 8 (21.6%), “face-to-face education”: 8 (21.6%), “both are fine”: 20 (54.1%), unanswered: 1 (2.7%). "both are fine" mainly was selected regardless of answers in Q1 or Q2 (Supplementary Table 5). One of the reasons for choosing “distance education” or “both are fine” (Question 12) was that the presentation materials were easy to read. Many of the reasons for choosing “face-to-face education” (Question 12) were that the facial expressions of presenters and teachers could not be seen remotely. This was pointed out by the students who chose “distance education”.

### Good points and improvements in the distance education (Q 13 and 14)

The good points (Question 13) were ease of participation, effective use of time, viewing recorded lectures, ease of group working online after classes, and explicit role sharing. There were some opinions that it was possible to plan tightly with a sense of crisis and share the roles clearly due to the difficulty of distance education.

Improvements (Question 14) included lack of casual communication with teachers, of casual use of Zoom after classes, of use of chat, and of time for group work, communication tools not being unified, and inability to grasp the progress of other groups. Some students were worried because they did not know when teachers would come to the breakout room.

## Discussion

Regarding communication between students and with teachers (Questions 1 and 2), although about 30% of the students felt difficulty, it was suggested that students could communicate with each other in remote group work. However, since some students felt difficulty, we should extract the problems from the free description of the questionnaire and consider how to deal with them.

In communication between students, there were two challenges on speaking, such as timing and reading intent and on sharing various information by an individual laptop. Answers to Questions 3, 4, and 5 could give some ideas for solutions for problems in communication between students. From the viewpoint of communication in remote group work using Zoom, it was suggested that the number of students in the group should be 4 or 5 in consideration of role sharing (Answers to Questions 4 and 5). However, even in group work with 4 or 5 students, the difficulty of speaking remained. Some solutions would be designating the speaker by the leader and making it easier for each student to understand the beginning and end of the statement. In addition, it would be important to see the faces of all the group members at the same time when discussing while sharing the screen with Zoom (Answers to Questions 3 and 5). A verbal explanation would not be sufficient because it is difficult to communicate based on common sense when the backgrounds of the students are diverse. Nonverbal communication that does not interrupt the explanation is important for facilitating the discussion. For example, it is possible to change the explanation based on the degree of understanding that can be seen from facial expressions. An et al. (An et al., 2008) pointed out the problem in remote group work was that nonverbal communication, including facial expressions, was not possible when there was no video conference tool. Other methods to further improve distance communication between students would be the use of devices such as a dual display for expanding the screen display and pen tabs for Zoom's whiteboard function. These make it possible to perform multiple tasks at the same time which are advantages of face-to-face education. However, it should be noted that under the present circumstances, many of the students cannot use them. In addition, it is difficult that one group is divided into smaller subgroups and discussions are held in the same space on Zoom. This means that it is difficult to realize detailed communication that can be realized in face-to-face education. Based on the student's environment, it is necessary to develop new communication methods.

When communicating with teachers, asking questions individually and the involvement of teachers in the breakout room were difficult. One way to solve the problems is to fix the time for teachers to go around the breakout room. However, if the discussion in one group does not converge, moving to other groups due to the time limit makes it difficult to correct the discussion with appropriate intervention. With the Zoom update in September 2020, all participants are able to select a breakout room and to see where teachers are (v.5.3.0 or later). Such technological innovations would solve problems. However, the goodness of face-to-face education, such as casual communication with teachers after class, remains lost. It should be noted that chat, email, and setting up another opportunity could not be fundamental solutions for this problem.

One of the challenges other than communication is the use of highly confidential data. As our PBL with pseudo data based on real consultation, sharing the data with the students was not a problem. When it is difficult to share highly confidential data, it is necessary to improve the environment for data sharing. An infrastructure environment, including communication networks, is important for the digitization of university education. In addition, supporting tools for programming and communication are important in distance education. In our PBL in 2020, many of the students had experience using R and RStudio, so there was no problem with programming. Communication tools such as Teams and Slack are useful for distance education but using various tools is bothersome. Considering the experiences of the students, it would be necessary to devise a manual for communications tools.

Future work is to evaluate the learning effect of synchronous distance education for PBL in data science education. Although there is room for improvement in this study, the feasibility of distance education was verified, so it is considered meaningful to compare the learning effects with face-to-face education. In 2019, the PBL with the same content was implemented as face-to-face education. Comparing the average scores by the teachers, the average score in 2019 (93.3 points) is higher than in 2020 (84.5 points). However, these scores cannot be simply compared because the number of students (20 in 2019) and the background of students were different, and the experience of teachers was accumulated. Since it is not clear how to evaluate the learning effect of PBL, research is needed in the future to consider how to evaluate the learning effect of face-to-face and distance education.

## Conclusion

In this study, we verified the feasibility and problems of distance education from the practice of synchronous distance education for PBL in data science education by questionnaire survey. As a result, it was found that although there are some issues to be improved, distance education for PBL could be sufficiently implemented based on our practice. Distance education for PBL in data science has a great advantage for future education regardless of the pandemic of COVID-19 because there is a shortage of data science teachers in Japan, and it is challenging to implement practical education such as PBL.

## Supplementary Information

Below is the link to the electronic supplementary material.

Supplementary file1 (DOCX 22 KB)
