# Supplementary material for: Experience of distance education for project-based learning in data science
Source: Jpn J Stat Data Sci. 2022 Apr 9;5(2):757–67. doi: 10.1007/s42081-022-00154-2 (PMC8994060; doi:10.1007/s42081-022-00154-2)
Supplement: Supplementary file 1 — Supplementary file1 (DOCX 22 KB) [file 42081_2022_154_MOESM1_ESM.docx]

Supplementary Table 1. Summary of Q1 and Q2.

|  | Q2 | easy | somewhat easy | neither easy  nor difficult | somewhat difficult | difficult | Total |
| --- | --- | --- | --- | --- | --- | --- | --- |
| Q1 |  |  |  |  |  |  |  |
| easy | | 4  (50.0%) | 2  (25.0%) | 2  (25.0%) | 0  (0%) | 0  (0%) | 8 |
| somewhat easy | | 0  (0%) | 7  (53.9%) | 4  (30.8%) | 2  (15.4%) | 0  (0%) | 13 |
| neither easy  nor difficult | | 0  (0%) | 1  (25.0%) | 0  (0%) | 3  (75.0%) | 0  (0%) | 4 |
| somewhat difficult | | 0  (0%) | 0  (0%) | 3  (37.5%) | 4  (50.0%) | 1  (12.5%) | 8 |
| difficult | | 0  (0%) | 1  (33.3%) | 0  (0%) | 1  (33.3%) | 1  (33.3%) | 3 |
| Total | | 4 | 11 | 9 | 10 | 2 | 36 |

Q1. Communication between students in discussions using Zoom

Q2. Communication with teachers in discussions using Zoom

Supplementary Table 2. Summaries of Q4 by Q1 and Q2

|  | Q4 | 2 | 3 | 4 | 5 | 6 | Total |
| --- | --- | --- | --- | --- | --- | --- | --- |
| Q1 |  |  |  |  |  |  |  |
| easy / somewhat easy | | 0  (0%) | 0  (0%) | 12  (57.1%) | 7  (33.3%) | 2  (9.5%) | 21 |
| neither easy nor difficult | | 0  (0%) | 1  (25.0%) | 2  (50.0%) | 1  (25.0%) | 0  (0%) | 4 |
| somewhat difficult / difficult | | 1  (9.1%) | 1  (9.1%) | 5  (45.5%) | 4  (36.4%) | 0  (0%) | 11 |
|  | | | | | | | |
|  | Q4 | 2 | 3 | 4 | 5 | 6 | Total |
| Q2 |  |  |  |  |  |  |  |
| easy / somewhat easy | | 0  (0%) | 0  (0%) | 10  (66.7%) | 4  (26.7%) | 1  (6.7%) | 15 |
| neither easy nor difficult | | 0  (0%) | 0  (0%) | 4  (44.4%) | 4  (44.4%) | 1  (11.1%) | 9 |
| somewhat difficult / difficult | | 1  (8.3%) | 2  (16.7%) | 5  (41.7%) | 4  (33.3%) | 0  (0%) | 12 |

Q1. Communication between students in discussions using Zoom

Q2. Communication with teachers in discussions using Zoom

Q4. What number of students is appropriate as one group in remote group work

Supplementary Table 3. Summaries of Q7 by Q1 and Q2

|  | Q7 | distance education | both are fine | face-to-face education | Total |
| --- | --- | --- | --- | --- | --- |
| Q1 |  |  |  |  |  |
| easy / somewhat easy | | 15  (71.4%) | 5  (23.8%) | 1  (4.8%) | 21 |
| neither easy nor difficult | | 1  (25.0%) | 2  (50.0%) | 1  (25.0%) | 4 |
| somewhat difficult / difficult | | 3  (27.3%) | 4  (36.4%) | 4  (36.4%) | 11 |
|  | | | | | |
|  | Q7 | distance education | both are fine | face-to-face education | Total |
| Q2 |  |  |  |  |  |
| easy / somewhat easy | | 9  (60.0%) | 5  (33.3%) | 1  (6.7%) | 15 |
| neither easy nor difficult | | 7  (77.8%) | 2  (22.2%) | 0  (0%) | 9 |
| somewhat difficult / difficult | | 3  (25.0%) | 4  (33.3%) | 5  (41.7%) | 12 |

Q1. Communication between students in discussions using Zoom

Q2. Communication between students in discussions using Zoom

Q7. Desirable style of lecture

Supplementary Table 4. Summaries of Q9 by Q1 and Q2

|  | Q9 | distance education | both are fine | face-to-face education | Total |
| --- | --- | --- | --- | --- | --- |
| Q1 |  |  |  |  |  |
| easy / somewhat easy | | 4  (19.1%) | 8  (38.1%) | 9  (42.9%) | 21 |
| neither easy nor difficult | | 0  (0%) | 0  (0%) | 4  (100%) | 4 |
| somewhat difficult / difficult | | 1  (9.1%) | 1  (9.1%) | 9  (81.8%) | 11 |
|  | | | | | |
|  | Q9 | distance education | both are fine | face-to-face education | Total |
| Q2 |  |  |  |  |  |
| easy / somewhat easy | | 1  (6.7%) | 5  (33.3%) | 9  (60.0%) | 15 |
| neither easy nor difficult | | 4  (44.4%) | 3  (33.3%) | 2  (22.2%) | 9 |
| somewhat difficult / difficult | | 0  (0%) | 1  (8.3%) | 11  (91.7%) | 12 |

Q1. Communication between students in discussions using Zoom

Q2. Communication between students in discussions using Zoom

Q9. Desirable style of group work

Supplementary Table 5. Summaries of Q11 by Q1 and Q2

|  | Q11 | distance education | both are fine | face-to-face education | Total |
| --- | --- | --- | --- | --- | --- |
| Q1 |  |  |  |  |  |
| easy / somewhat easy | | 7  (33.3%) | 11  (52.4%) | 3  (14.3%) | 21 |
| neither easy nor difficult | | 0  (0%) | 3  (75.0%) | 1  (25.0%) | 4 |
| somewhat difficult / difficult | | 1  (9.1%) | 6  (54.6%) | 4  (36.4%) | 11 |
|  | | | | | |
|  | Q11 | distance education | both are fine | face-to-face education | Total |
| Q2 |  |  |  |  |  |
| easy / somewhat easy | | 3  (20.0%) | 11  (73.3%) | 1  (6.7%) | 15 |
| neither easy nor difficult | | 4  (44.4%) | 3  (33.3%) | 2  (22.2%) | 9 |
| somewhat difficult / difficult | | 1  (8.3%) | 6  (50.0%) | 5  (41.7%) | 12 |

Q1. Communication between students in discussions using Zoom

Q2. Communication between students in discussions using Zoom

Q11. Desirable style of presentation
